# Supplementary material for: Cryo-EM structure of the transposon-associated TnpB enzyme
Source: Nature. 2023 Apr 5;616(7956):390–7. doi: 10.1038/s41586-023-05933-9 (PMC10097598; doi:10.1038/s41586-023-05933-9)
Supplement: Supplementary file 1 — This file contains the following: Supplementary Fig. 1: Uncropped images of SDS–PAGE and Urea-PAGE gels. Supplementary Fig. 2: Uncropped images of northern blotting analysis. Supplementary Table 1: Nucleic acid sequences used in this study. Supplementary Table 2: Primers and constructs in this study. [file 41586_2023_5933_MOESM1_ESM.pdf]

nature portfolio

https://doi.org/10.1038/s41586-023-05933-9

Supplementary information

Cryo-EM structure of the transposon-associated TnpB enzyme

In the format provided by the authors and unedited

Supplementary Information for

Cryo-EM structure of the transposon-associated TnpB enzyme

Ryoya Nakagawa[1,11], Hisato Hirano[1,11], Satoshi N. Omura[1], Suchita Nety[2,3,4,5,6], Soumya Kannan[2,3,4,5,6], Han Altae-Tran[2,3,4,5,6], Xiao Yao[7], Yuriko Sakaguchi[7], Takayuki Ohira[7], Wen Y. Wu[8], Hiroshi Nakayama[9], Yutaro Shuto[1], Tatsuki Tanaka[1], Fumiya K. Sano[1], Tsukasa Kusakizako[1], Yoshiaki Kise[1,10], Yuzuru Itoh[1], Naoshi Dohmae[9], John van der Oost[8], Tsutomu Suzuki[7], Feng Zhang[2,3,4,5,6], and Osamu Nureki[1,10,*]

*Correspondence: nureki@bs.s.u-tokyo.ac.jp

This PDF file includes:

Supplementary Figs. 1, 2
Supplementary Tables 1, 2

a

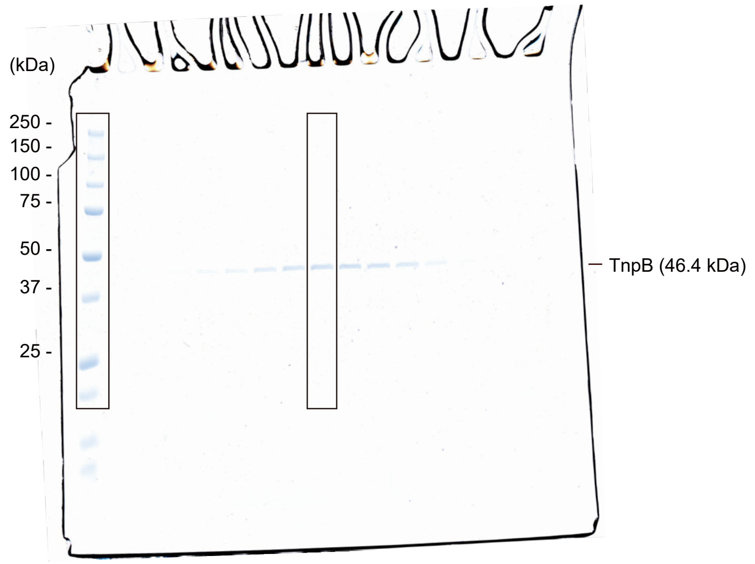

b

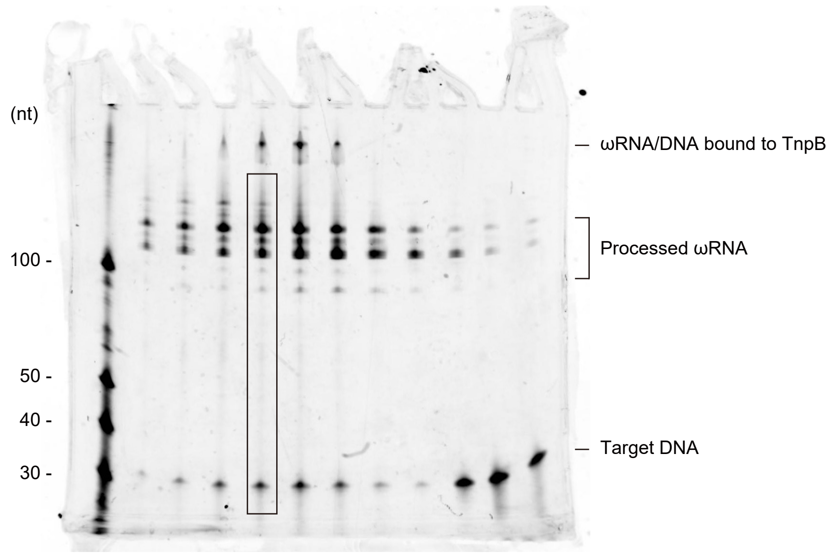

Supplementary Fig. 1. Uncropped images of SDS-PAGE and Urea-PAGE gels.
a, SDS-PAGE gel of SEC peak fraction (used for Extended Data Fig. 2a).
b, Urea-PAGE gel of SEC peak fraction (used for Extended Data Fig. 2a).

a

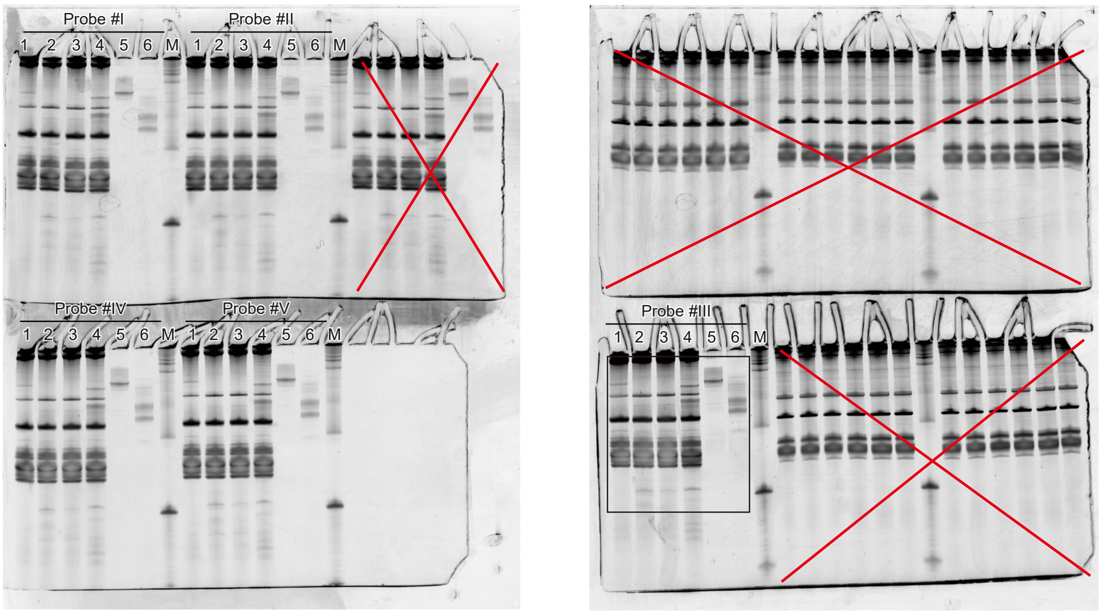

b

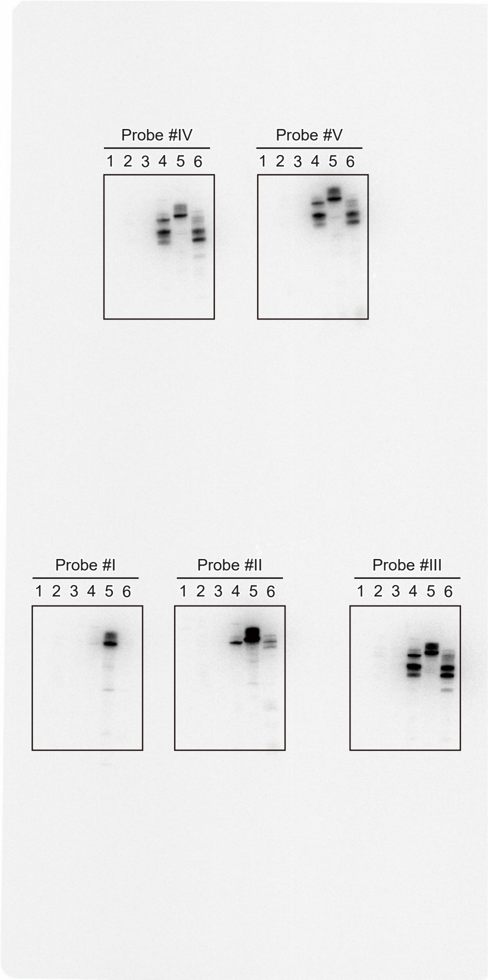

Supplementary Fig. 2. Uncropped images of northern blotting analysis.
a, Uncropped 10% gel polyacrylamide gel visualized with GelGreen.
b, Uncropped membrane used for Extended Data Fig. 5c.

Supplementary Table 1. Nucleic-acid sequences used in this study.

cryo-EM analysis

Name	Sequence
ωRNA	GAUUCAAGAAUCCCGAAGUGAAGAAUCUUGCCGUCCGUACAUGGACUUGCCCGAACUGUGGGGAAACCCAUGACCGAGACGAGAACGCUGC<br>GCUGAACAUUCGGCGUGAAGCGUUGGUGGCUGCGGGAAUCUCAGACACCUUAAACGCUCAUGGAGGCUAUGUCAGACCUGCUUCGGCGGG<br>CAAUGGUCUGCGAAGUGAGAAUCACGCGACUUUAGUCGUGUGAGGUUCAAGAGUCCCUUGGCGCCC
TS	A*T*T*T*G*A*A*T*GGGCGCCAAGGGACTCATCAATAGAAA
NTS	TTTCTATTGATGAGTCCCTTGG*C*G*C*C*C*A*T*T*C*A*A*A*T*

The guide sequence in the wRNA and the target sequence in the TS and NTS are underlined. The TAM sequence (TTGAT) in the NTS is colored purple. * phosphorothioate bond.

In vitro DNA cleavage assays

Name	Sequence
Target DNA	AGCGCCCAATACGCAAACCGCCTCTCCCCGCGCGTTGGCCGATTCATTAATGCAGCTGGCACGACAGGTTTCCCGACTGGAAAGCGGGCAGTG<br>AGCGCAACGCAATTAATGTGAGTTAGCTCACTCATTAGGCACCCCAGGCTTTACACTTTATGCTTCCGGCTCGTATGTTGTGTGGAATTGTGAGC<br>GGATAACAATTTCACACAGGAAACAGCTATGACCATGATTACGCCAAGCTTGCATGCCTGCAGGTCGACTCTAGAGGATCCCCGGGTACCGAGC<br>TCGAATTCACTGGCCGTCGTTTTACAACGTCGTGACTGGGAAAACCCTGGCGTTACCCAACTTAATCGCCTTGCAGCACATCCCCCTTTCGCCA<br>GCTGGCGTAATAGCGAAGAGGCCCGCACCGATCGCCCTTCCCAACAGTTGCGCAGCCTGAATGGCGAATGGCGCCTGATGCGGTATTTTCTCC<br>TTACGCATCTGTGCGGTATTTCACACCGCATACGTCAAAGCAACCATAGTACGCGCCCTGTAGCGGCGCATTAAGCGCGGCGGGTGTGGTGGT<br>TACGCGCAGCGTGACCGCTACACTTGCCAGCGCCCTAGCGCCCGCTCCTTTCGCTTTCTTCCCTTCCTTTCTCGCCACGTTCGCCGGCTTTCCC<br>CGTCAAGCTCTAAATCGGGGGCTCCCTTTAGGGTTCCGATTTAGTGCTTTACGGCACCTCGACCCCAAAAAACTTGATTTGGGTGATGGTTCAC<br>GTAGTGGGCCATCGCCCTGATAGACGGTTTTTCGCCCTTTGACGTTGGAGTCCACGTTCTTTAATAGTGGACTCTTGTTCCAAACTGGAACAACA<br>CTCAACCCTATCTCGGGCTATTCTTTTGATTTATAAGGGATTTTGCCGATTTCGGCCTATTGGTTAAAAAATGAGCTGATTTAACAAAAATTTAACG<br>CGAATTTTAACAAAATATTAACGTTTACAATTTTATGGTGCACTCTCAGTACAATCTGCTCTGATGCCGCATAGTTAAGCCAGCCCCGACACCCGC<br>CAACACCCGCTGACGCGCCCTGACGGGCTTGTCTGCTCCCGGCATCCGCTTACAGACAAGCTGTGACCGTCTCCGGGAGCTGCATGTGTCAGA<br>GGTTTTCACCGTCATCACCGAAACGCGCGAGACGAAAGGGCCTCGTGATACGCCTATTTTTATAGGTTAATGTCATGATAATAATGGTTTCTTAG<br>ACGTCAGGTGGCACTTTTCGGGGAAATGTGCGCGGAACCCCTATTTGTTTATTTTTCTATTGATGAGTCCCTTGGCGCCCATTCAAATATGTATC<br>CGCTCATGAGACAATAACCCTGATAAATGCTTCAATAATATTGAAAAAGGAAGAGTATGAGTATTCAACATTTCCGTGTCGCCCTTATTCCCTTTTT<br>TGCGGCATTTTGCCTTCCTGTTTTTGCTCACCCAGAAACGCTGGTGAAAGTAAAAGATGCTGAAGATCAGTTGGGTGCACGAGTGGGTTACATC<br>GAACTGGATCTCAACAGCGGTAAGATCCTTGAGAGTTTTCGCCCCGAAGAACGTTTTCCAATGATGAGCACTTTTAAAGTTCTGCTATGTGGCGC<br>GGTATTATCCCGTATTGACGCCGGGCAAGAGCAACTCGGTCGCCGCATACACTATTCTCAGAATGACTTGGTTGAGTACTCACCAGTCACAGAA<br>AAGCATCTTACGGATGGCATGACAGTAAGAGAATTATGCAGTGCTGCCATAACCATGAGTGATAACACTGCGGCCAACTTACTTCTGACAACGAT<br>CGGAGGACCGAAGGAGCTAACCGCTTTTTTGCACAACATGGGGGATCATGTAACTCGCCTTGATCGTTGGGAACCGGAGCTGAATGAAGCCAT<br>ACCAAACGACGAGCGTGACACCACGATGCCTGTAGCAATGGCAACAACGTTGCGCAAACTATTAACTGGCGAACTACTTACTCTAGCTTCCCGG<br>CAACAATTAATAGACTGGATGGAGGCGGATAAAGTTGCAGGACCACTTCTGCGCTCGGCCCTTCCGGCTGGCTGGTTTATTGCTGATAAATCTG<br>GAGCCGGTGAGCGTGGGTCTCGCGGTATCATTGCAGCACTGGGGCCAGATGGTAAGCCCTCCCGTATCGTAGTTATCTACACGACGGGGAGT<br>CAGGCAACTATGGATGAACGAAATAGACAGATCGCTGAGATAGGTGCCTCACTGATTAAGCATTGGTAACTGTCAGACCAAGTTTACTCATATAT<br>ACTTTAGATTGATTTAAAACTTCATTTTTAATTTAAAAGGATCTAGGTGAAGATCCTTTTTGATAATCTCATGACCAAAATCCCTTAACGTGAGTTTT<br>CGTTCCACTGAGCGTCAGACCCCGTAGAAAAGATCAAAGGATCTTCTTGAGATCCTTTTTTTCTGCGCGTAATCTGCTGCTTGCAAACAAAAAAA<br>CCACCGCTACCAGCGGTGGTTTGTTTGCCGGATCAAGAGCTACCAACTCTTTTTCCGAAGGTAACTGGCTTCAGCAGAGCGCAGATACCAAATA<br>CTGTCCTTCTAGTGTAGCCGTAGTTAGGCCACCACTTCAAGAACTCTGTAGCACCGCCTACATACCTCGCTCTGCTAATCCTGTTACCAGTGGCT<br>GCTGCCAGTGGCGATAAGTCGTGTCTTACCGGGTTGGACTCAAGACGATAGTTACCGGATAAGGCGCAGCGGTCGGGCTGAACGGGGGGTTC<br>GTGCACACAGCCCAGCTTGGAGCGAACGACCTACACCGAACTGAGATACCTACAGCGTGAGCTATGAGAAAGCGCCACGCTTCCCGAAGGGA<br>GAAAGGCGGACAGGTATCCGGTAAGCGGCAGGGTCGGAACAGGAGAGCGCACGAGGGAGCTTCCAGGGGGAAACGCCTGGTATCTTTATAGT<br>CCTGTCGGGTTTCGCCACCTCTGACTTGAGCGTCGATTTTTGTGATGCTCGTCAGGGGGGCGGAGCCTATGGAAAAACGCCAGCAACGCGGCC<br>TTTTTACGGTTCCTGGCCTTTTGCTGGCCTTTTGCTCACATGTTCTTTCCTGCGTTATCCCCTGATTCTGTGGATAACCGTATTACCGCCTTTGAG<br>TGAGCTGATACCGCTCGCCGCAGCCGAACGACCGAGCGCAGCGAGTCAGTGAGCGAGGAAGCGGAAG
Target DNA-MM1/2	~~GTCAGGTGGCACTTTTCGGGGAAATGTGCGCGGAACCCCTATTTGTTTATTTTTCTATTGATCTGTCCCTTGGCGCCCATTCAAATATGTAT~~
Target DNA-MM3/4	~~GTCAGGTGGCACTTTTCGGGGAAATGTGCGCGGAACCCCTATTTGTTTATTTTTCTATTGATCTCACCCTTGGCGCCCATTCAAATATGTAT~~
Target DNA-MM5/6	~~GTCAGGTGGCACTTTTCGGGGAAATGTGCGCGGAACCCCTATTTGTTTATTTTTCTATTGATCTGTGGCTTGGCGCCCATTCAAATATGTAT~~
Target DNA-MM7/8	~~GTCAGGTGGCACTTTTCGGGGAAATGTGCGCGGAACCCCTATTTGTTTATTTTTCTATTGATCTGTCCGATGGCGCCCATTCAAATATGTAT~~
Target DNA-MM9/10	~~GTCAGGTGGCACTTTTCGGGGAAATGTGCGCGGAACCCCTATTTGTTTATTTTTCTATTGATCTGTCCCTACGCGCCCATTCAAATATGTAT~~
Target DNA-MM11/12	~~GTCAGGTGGCACTTTTCGGGGAAATGTGCGCGGAACCCCTATTTGTTTATTTTTCTATTGATCTGTCCCTTGCGGCCCATTCAAATATGTAT~~
Target DNA-MM13/14	~~GTCAGGTGGCACTTTTCGGGGAAATGTGCGCGGAACCCCTATTTGTTTATTTTTCTATTGATCTGTCCCTTGGCCGCCATTCAAATATGTAT~~
Target DNA-MM15/16	~~GTCAGGTGGCACTTTTCGGGGAAATGTGCGCGGAACCCCTATTTGTTTATTTTTCTATTGATCTGTCCCTTGGCGCGGATTCAAATATGTAT~~
Target DNA-1 (TG1)	~~GTCAGGTGGCACTTTTCGGGGAAATGTGCGCGGAACCCCTATTTGTTTATTTTTCTATTGATCTGTCCCTTGGCGCGGCGCGCGATATGTAT~~
Target DNA-2 (TG2)	~~GTCAGGTGGCACTTTTCGGGGAAATGTGCGCGGAACCCCTATTTGTTTATTTTTCTATTGATCTGTCCCTTGGCGCGGATCGCGATATGTAT~~
Target DNA-3 (TG3)	~~GTCAGGTGGCACTTTTCGGGGAAATGTGCGCGGAACCCCTATTTGTTTATTTTTCTATTGATCTGTCCCTTGGCGCGGATATCGATATGTAT~~
Target DNA-4 (TG4)	~~GTCAGGTGGCACTTTTCGGGGAAATGTGCGCGGAACCCCTATTTGTTTATTTTTCTATTGATCTGTCCCTTGGCGCGGATTCAAATATGTAT~~
Target DNA-5 (TG5)	~~GTCAGGTGGCACTTTTCGGGGAAATGTGCGCGGAACCCCTATTTGTTTATTTTTCTATTGATCTGTCCCTTGGCGCGGATATATATATGTAT~~

The target sequence are underlined. The TAM sequence (TTGAT) is colored purple. Mismatches, CG, and AT are colored red, blue, and green, respectively.
Target DNA-MM12–Target DNA-ATATAT are shown only around the target sequence.

Genome editing assay

Site #	Gene	Guide sequence
1	AGBL1	AATGAATGGCTACTCTAACC
2	EMX1	GTGATGGGAGCCCTTCTTCT
3	AGBL1	GATTAATAAGTATCTTGTGG
4	DYNC1H1	TTCAAATCAAGCCACTTTTG
5	HPRT1	GTAATCCAGCAGGTCAGCAA
6	VEGFA	GTCAGCTAATTCTGACTCCT
7	EMX1	TTAGATTATGCATATACCAG

Supplementary Table 2. Primers and constructs used in this study.

TnpB mutants

Mutation	Forward primer	Reverse primer
ΔCTD	GTTGGTGGCTTAACGGCCACGCGATCGCTG	GTGGCCGTTACGCAGCCACCAACGCTTCAC
Y52A	CTGACCGCCGGGCAAACGAGTAGCGAACTG	TTGCCCGGCGGTCAGTCCCTTCCCGCTTTC
S56A	GCCAGCGAACTGACCCTTCTGAAGCAG	CGTTTGCCCGTAGGTCAGTCCC
K76A	GCCTTTGCTTTGCAGAACTCGTGAAAAAC	ATCTACTTCCGAGAGCCAGGAGGTTTC
F77A	GCCGCTTTGCAGAACTCGCTGAAAAACC	CTTATCTACTTCCGAGAGCCAGGAGG
Q80A	GTTTGCTTTGGCCAACTCGCTGAAAACC	TCAGCGAGTTGGCCAAAGCAAACTTATCTAC
T123A	GACTCAATTCGCCAACAACAACATCCAAATTG	TGTTGTTGTTGGCGAATTGAGTCCGGTAG
N124A	TCAATTCACCGCCAACAACATCCAAATTGG	GGATGTTGTTGGCGGTGAATTGAGTCCG

Target DNA mutation

Mutation	Forward primer	Reverse primer
Target DNA-MM1/2	CTGTCCCTTGGCGCCCATTCAAATATG	ATCAATAGAAAAATAAACAAATAGGGGTTCCGCG
Target DNA-MM3/4	CACCCTTGGCGCCCATTCAAATATGTATC	TCATCAATAGAAAAATAAACAAATAGGGGTTCCGC
Target DNA-MM5/6	GGCTTGGCGCCCATTCAAATATGTATCCG	ACTCATCAATAGAAAAATAAACAAATAGGGGTTCC
Target DNA-MM7/8	GATGGCGCCCATTCAAATATGTATCCG	GGACTCATCAATAGAAAAATAAACAAATAGGGG
Target DNA-MM9/10	ACGCGCCCATTCAAATATGTATCCGCTC	AGGGACTCATCAATAGAAAAATAAACAAATAGGGG
Target DNA-MM11/12	CGGCCCATTCAAATATGTATCCGCTCATGAG	CAAGGGACTCATCAATAGAAAAATAAACAAATAGG
Target DNA-MM13/14	CGCCATTCAAATATGTATCCGCTCATGAGAC	GCCAAGGGACTCATCAATAGAAAAATAAAC
Target DNA-MM15/16	GGATTCAAATATGTATCCGCTCATGAGACAATAAC	GCGCCAAGGGACTCATCAATAGAAAAATAAAC
Target DNA-1 (TG1)	CGCGCGATATGTATCCGCTCATGAGACAATAACCC	GGGCGCCAAGGGACTCATCAATAG
Target DNA-2 (TG2)	ATCGCGATATGTATCCGCTCATGAGACAATAACCC	GGGCGCCAAGGGACTCATCAATAG
Target DNA-3 (TG3)	ATATCGATATGTATCCGCTCATGAGACAATAACCC	GGGCGCCAAGGGACTCATCAATAG
Target DNA-5 (TG5)	ATATATATATGTATCCGCTCATGAGACAATAACCC	GGGCGCCAAGGGACTCATCAATAG

DNA probes used in the northern blotting experiment

Probe number	Oligo sequence	Reverse primer
Probe #I	TTCTTCACTTCGGGATTCTTGAATC	ATCAATAGAAAAATAAACAAATAGGGGTTCCGCG
Probe #II	CGTCTCGGTCATGGGTTTCCCCACA	TCATCAATAGAAAAATAAACAAATAGGGGTTCCGC
Probe #III	GTCTGAGATTCCCGCAGCCACCAAC	ACTCATCAATAGAAAAATAAACAAATAGGGGTTCC
Probe #IV	GCAGACCATTGCCCGCCGAAGCAGG	GGACTCATCAATAGAAAAATAAACAAATAGGGG
Probe #V	GGGCGCCAAGGGACTCTTGAACCTC	AGGGACTCATCAATAGAAAAATAAACAAATAGGGG

Genome editing assay

Site #	Gene	Guide sequence	Reverse primer
1	AGBL1	CAGCATGTTCCTCACAAAGAGAGTCTAC	TGCAAAGCCATTATTGCACTTGGAGAG
2	EMX1	CAGCTCAGCCTGAGTGTTGA	CTCGTGGGTTTGTGGTTGC
3	AGBL1	GACTGTCCCATTACTGGGGATGTTAACTTT	TGCCTGAGGCAGAGCAGCA
4	DYNC1H1	GACTAGCCTTGGTCTGGGTGGAATG	AACTAATTTCCTGTAAGGCAGTTCCTGATGC
5	HPRT1	CATCACATTGTAGCCCTCTGTGTGC	CCACACACACCTGTAAAATAAGTATATATCCTCCAAG
6	VEGFA	CCCACCACCTTCTCAGCTCATGT	TAACGTCAACCATACCAACAGCTGTTTAT
7	EMX1	CAGTCATTCCCCTTGCTCTCTGCT	CCCAGGGCTCTTCCCTCATCTC

Construct

Plasmid	
pETDuet-ISDra2TnpB-ωRNA	https://benchling.com/s/seq-rK3nFj2GszhltgQ1YtfI?m=slm-ojgLECTb9cfZAmxu8knO
pHS1288	human codon optimized ISDra2 TnpB protein expression
pHS1201	human U6 promoter ISDra2 scaffold from Karvelis et al with BpiI Golden Gate site
SN0856	human U6 promoter ISDra2 trim1 scaffold with BpiI Golden Gate site
SN0857	human U6 promoter ISDra2 trim2 scaffold with BpiI Golden Gate site
SN1338	human U6 promoter ISDra2 trim3 scaffold with BpiI Golden Gate site
