# Supplementary material for: Cryo-EM structure of the transposon-associated TnpB enzyme
Source: Nature. 2023 Apr 5;616(7956):390–7. doi: 10.1038/s41586-023-05933-9 (PMC10097598; doi:10.1038/s41586-023-05933-9)
Supplement: Supplementary file 2 — Reporting Summary [file 41586_2023_5933_MOESM2_ESM.pdf]

nature portfolio

Corresponding author(s): Osamu Nureki

Last updated by author(s): Feb 10, 2023

Reporting Summary

Nature Portfolio wishes to improve the reproducibility of the work that we publish. This form provides structure for consistency and transparency in reporting. For further information on Nature Portfolio policies, see our Editorial Policies and the Editorial Policy Checklist.

Statistics

For all statistical analyses, confirm that the following items are present in the figure legend, table legend, main text, or Methods section.

n/a	Confirmed	
☐	☒	The exact sample size (*n*) for each experimental group/condition, given as a discrete number and unit of measurement
☐	☒	A statement on whether measurements were taken from distinct samples or whether the same sample was measured repeatedly
☒	☐	The statistical test(s) used AND whether they are one- or two-sided<br>*Only common tests should be described solely by name; describe more complex techniques in the Methods section.*
☒	☐	A description of all covariates tested
☒	☐	A description of any assumptions or corrections, such as tests of normality and adjustment for multiple comparisons
☐	☒	A full description of the statistical parameters including central tendency (e.g. means) or other basic estimates (e.g. regression coefficient) AND variation (e.g. standard deviation) or associated estimates of uncertainty (e.g. confidence intervals)
☒	☐	For null hypothesis testing, the test statistic (e.g. F, t, r) with confidence intervals, effect sizes, degrees of freedom and P value noted<br>*Give P values as exact values whenever suitable.*
☒	☐	For Bayesian analysis, information on the choice of priors and Markov chain Monte Carlo settings
☒	☐	For hierarchical and complex designs, identification of the appropriate level for tests and full reporting of outcomes
☒	☐	Estimates of effect sizes (e.g. Cohen's *d*, Pearson's *r*), indicating how they were calculated

Our web collection on statistics for biologists contains articles on many of the points above.

Software and code

Policy information about availability of computer code

Data collection	EPU (version 2.12)
Data analysis	cryoSPARC (version 3.3.2), COOT (version 0.9), UCSF ChimeraX (version 1.1.1), CueMol2 (http://www.cuemol.org/ version 2.2.3.443), PHENIX (version 1.20.1).

For manuscripts utilizing custom algorithms or software that are central to the research but not yet described in published literature, software must be made available to editors and reviewers. We strongly encourage code deposition in a community repository (e.g. GitHub). See the Nature Portfolio guidelines for submitting code & software for further information.

Data

Policy information about availability of data

All manuscripts must include a data availability statement. This statement should provide the following information, where applicable:

- Accession codes, unique identifiers, or web links for publicly available datasets
- A description of any restrictions on data availability
- For clinical datasets or third party data, please ensure that the statement adheres to our policy

The atomic models have been deposited in the Protein Data Bank under the accession code 8H1J. The cryo-EM density map has been deposited in the Electron Microscopy Data Bank under the accession code EMD-34428.

Human research participants

Policy information about studies involving human research participants and Sex and Gender in Research.

Reporting on sex and gender	n/a
Population characteristics	n/a
Recruitment	n/a
Ethics oversight	n/a

Note that full information on the approval of the study protocol must also be provided in the manuscript.

Field-specific reporting

Please select the one below that is the best fit for your research. If you are not sure, read the appropriate sections before making your selection.

☒ Life sciences ☐ Behavioural & social sciences ☐ Ecological, evolutionary & environmental sciences

For a reference copy of the document with all sections, see nature.com/documents/nr-reporting-summary-flat.pdf

Life sciences study design

All studies must disclose on these points even when the disclosure is negative.

Sample size	No statistical method was used to determine the sample size. For cryo-EM analyses, sample sizes were determined by the availability of microscope time and the number of particles on electron microscopy grids enough to obtain a structure at the reported resolution. For biochemical analysis, sample size were determined based on the previous reports of this type of study (Kato et al., Nat. Commun. 2022) and the reproducibility of results across independent experiments.
Data exclusions	For cryo-EM analyses, particles that did not contribute to improving map quality were excluded following the standard classification procedures in cryoSPARC. This is standard practice for structure determination by cryo-EM. For biochemical analyses, no data was excluded.
Replication	Biochemical experiments were performed at least three times.
Randomization	For cryo-EM analyses, particles were randomly assigned to half-maps for resolution determination following the standard procedures in cryoSPARC. For biochemical analyses, randomization is not relevant to this study, since no experimental group was assigned in all experiments.
Blinding	Blinding is not applicable to this study, since neither structural nor functional experiments included subjective assignments.

Reporting for specific materials, systems and methods

We require information from authors about some types of materials, experimental systems and methods used in many studies. Here, indicate whether each material, system or method listed is relevant to your study. If you are not sure if a list item applies to your research, read the appropriate section before selecting a response.

Materials & experimental systems

n/a	Involved in the study	
☒	☐	Antibodies
☐	☒	Eukaryotic cell lines
☒	☐	Palaeontology and archaeology
☒	☐	Animals and other organisms
☒	☐	Clinical data
☒	☐	Dual use research of concern

Methods

n/a	Involved in the study	
☒	☐	ChIP-seq
☒	☐	Flow cytometry
☒	☐	MRI-based neuroimaging

Eukaryotic cell lines

Policy information about cell lines and Sex and Gender in Research

Cell line source(s)	HEK293FT cell line was obtained from ThermoFisher Scientific (R70007).
Authentication	The HEK293FT cell line was not authenticated as it was purchased commercially.
Mycoplasma contamination	The HEK293FT cell line was not tested for mycoplasma contamination.
Commonly misidentified lines (See ICLAC register)	HEK293FT is not in the ICLAC database of misidentified cell lines.
